# Rethinking Vaginal Microbiome Resilience: A Conceptual Multi-Omic Framework

**DOI:** 10.3390/microorganisms14071536

**Published:** 2026-07-14

**Authors:** Brittnee Cagle-White, Rob E. Carpenter, Alaina Vincent, Ellen Kominek, Andrew Krouse

**Affiliations:** 1 School of Medicine, The University of Texas at Tyler, 3900 University Boulevard, Tyler, TX 75799, USA; 2 Soules College of Business, The University of Texas at Tyler, 3900 University Boulevard, Tyler, TX 75799, USA; 3 OSPRI BioPath, 7290 Virginia Parkway, Suite 3400, McKinney, TX 75071, USA; 4 Business Operations & Facilities, The University of Texas Medical Branch, 301 University Boulevard, Galveston, TX 77555, USA

**Keywords:** vaginal microbiome, genetic–hormonal interface, estrogen–glycogen axis, *Lactobacillus* dominance, bacterial vaginosis, *Gardnerella* diversity, host–microbe interactions

## Abstract

The vaginal microbiome is often interpreted through static taxonomic patterns. Yet microbial composition alone does not explain why some communities resist perturbation, recover after disruption, or transition toward dysbiosis. This narrative review synthesizes evidence that vaginal microbiome stability is shaped by endocrine phase, epithelial substrate availability, microbial functional capacity, mucosal tone and candidate host modifiers. High-estrogen states, particularly pregnancy, are associated with epithelial maturation, glycogen accumulation, low vaginal pH, and *Lactobacillus*-dominant communities, whereas postpartum, lactational, menopausal, and other hypoestrogenic states are associated with reduced epithelial support and increased vulnerability to diverse anaerobe-rich configurations. We review the linking of the estrogen–glycogen–*Lactobacillus* axis, focusing on microbial functions involved in glycogen degradation, lactate production and biofilm persistence, and host pathways that may modify mucosal responsiveness. Direct human genotype-to-vaginal-microbiome stability evidence remains limited; therefore, host genetic features are treated as candidate modifiers rather than validated clinical predictors. We propose a conceptual multi-omic hierarchy for organizing endocrine, epithelial, microbial, immune, temporal, and candidate host-modifier domains relevant to vaginal microbiome resilience. This framework is hypothesis-generating and requires longitudinal, phase-resolved human validation before quantitative prediction or clinical application.

## 1. Introduction

The vaginal microbiome (VMB) is an active ecological system shaped by hormonal signaling, host factors, and microbial adaptation [1,2]. Historically, clinical attention to vaginal dysbiosis, such as bacterial vaginosis (BV) or vulvovaginal candidiasis, has focused primarily on microbial taxa (e.g., overgrowth of anaerobes and decline of *Lactobacillus* species) [1,2]. However, this taxon-centric view underemphasizes the host and endocrine contexts that regulate microbial habitat and resilience [1,2]. Pregnancy and lactation provide physiological contexts for examining how the VMB remodels under hormone-driven mucosal changes and how instability may contribute to dysbiosis [3,4].

During pregnancy, the VMB is typically more stable, less diverse (i.e., lower alpha diversity), and dominated by *Lactobacillus* species [2,3,4]. In contrast, the immediate postpartum period is marked by rapid declines in estradiol and progesterone, mucosal remodeling, and an associated shift toward higher diversity and increased instability, irrespective of the preceding pregnancy community state type [4]. These transitions suggest that hormonal states may shape microbial composition and also ecological trajectories, including community resilience, redundancy, and susceptibility to perturbation [5,6].

From an ecological perspective, dysbiosis may reflect reduced community stability, diminished dominance of keystone taxa (e.g., *Lactobacillus crispatus*), and increased richness of generalist or opportunistic taxa [2,7,8]. Elevated vaginal microbial diversity has been associated with adverse outcomes, including preterm birth, ascending infection, and recurrent dysbiosis [7,8]; *L. crispatus* has also shown antimicrobial activity against preterm birth-associated vaginal bacteria in vitro [9]. Accordingly, the VMB may be conceptualized as an ecological system with tipping-point dynamics: stable when host and hormonal conditions favor keystone-taxon dominance, but vulnerable when hormonal, microbial, or other host-associated perturbations push it toward a high-diversity, low-resilience state [5,6].

These exemplars point to the genetic–hormonal interface in which variation in hormone-receptor signaling, glycogen metabolism, and epithelial or immune responses may influence mucosal responses to hormonal change [10,11]. Such responses may alter substrate availability, pH, and immune conditions associated with microbiome composition [10,11]. For example, a high-estradiol pregnancy environment may promote glycogen accumulation and *Lactobacillus* dominance [12]. Although relevant variants have been characterized in other tissues or systemic phenotypes [13,14,15], whether they modify vaginal glycogen availability or VMB resilience remains unproven in humans. Similarly, postpartum gonadal-steroid withdrawal may challenge microbial stability [4], but genotype-specific buffering has not been established. Integrating endocrine phase, candidate host modifiers, and microbial community structure may therefore help conceptualize vaginal health and dysbiosis as dynamic ecological states within a hormonally structured landscape [2,4]. This framework is hypothesis-generating and requires longitudinal human validation before it can support risk stratification, intervention, or personalized monitoring.

The purpose of this narrative review is to synthesize evidence on how hormonal transitions reshape the VMB, evaluate for candidate host genetic modifiers of mucosal responsiveness, examine microbial functional adaptations within hormone-structured vaginal niches, and propose a conceptual multi-omic framework for organizing microbiome stability at the genetic–hormonal interface.

## 2. Methods

### 2.1. Review Design

This paper was designed as a narrative review and conceptual synthesis rather than a systematic review or meta-analysis. Its purpose was to integrate evidence from reproductive microbiology, endocrine physiology, host genetics, microbial genomics, mucosal immunology, and systems biology to examine VMB stability as a dynamic phenotype. Because the review aimed to develop a mechanistic conceptual framework rather than estimate pooled effects, the literature was evaluated for conceptual relevance, biological plausibility, study design, and contribution to understanding host–endocrine–microbial determinants of vaginal ecological stability. The synthesis was not intended to assign quantitative weights, effect sizes, or clinical validity to individual candidate features.

### 2.2. Literature Search Strategy

A structured narrative search was conducted across PubMed/MEDLINE, Scopus, Web of Science, and Google Scholar to identify peer-reviewed studies relevant to VMB stability, hormonal regulation, glycogen metabolism, *Lactobacillus* dominance, BV, host genetic variation, microbial functional genomics, mucosal immunity, and predictive multi-omic modeling. Search terms were combined using Boolean operators and included variations of the following terms: “vaginal microbiome,” “vaginal microbiota,” “microbiome stability,” “bacterial vaginosis,” “*Lactobacillus crispatus*,” “*Lactobacillus iners*,” “estrogen,” “estradiol,” “progesterone,” “pregnancy,” “postpartum,” “lactation,” “menopause,” “glycogen,” “vaginal pH,” “lactic acid,” “ESR1,” “ESR2,” “host genetics,” “single nucleotide polymorphism,” “Toll-like receptor,” “cytokine,” “mucosal immunity,” “*Gardnerella*,” “*Prevotella*,” “microbial genomics,” “metagenomics,” “multi-omics,” “systems biology,” and “predictive modeling.”

Searches prioritized literature published in English and emphasized human studies when available. Foundational mechanistic studies, animal models, in vitro studies, genomic analyses, and systems-biology papers were included when they clarified biological pathways that could not be adequately described using human observational evidence alone. Reference lists of highly relevant reviews and primary studies were also examined to identify additional sources. Searches were conducted from database inception through March 2026 with emphasis on peer-reviewed English-language studies; because this was a narrative review, formal PRISMA screening counts and risk-of-bias scoring were not performed.

### 2.3. Inclusion and Exclusion Criteria

Studies were considered eligible for inclusion if they addressed at least one of the following domains: (a) VMB composition or stability across reproductive phases; (b) hormonal regulation of vaginal epithelial structure, glycogen availability, pH, or *Lactobacillus* dominance; (c) microbial genetic or metabolic programs involved in glycogen degradation, lactate production, mucin utilization, biofilm formation, or dysbiosis-associated persistence; (d) host genetic variation affecting endocrine signaling, glycogen metabolism, epithelial barrier function, innate immune recognition, cytokine regulation, or antimicrobial defense; or (e) computational, systems-biology, or multi-omic approaches relevant to modeling microbiome stability or ecological state transitions.

Studies were excluded when they focused only on unrelated microbiome sites, did not address host–microbe or endocrine–microbe mechanisms, lacked relevance to vaginal ecological stability, or provided only general commentary without mechanistic or empirical contribution. Studies of urinary, gastrointestinal, or systemic microbiomes were included only when they contributed a transferable methodological or systems-biology principle relevant to multi-omic integration.

### 2.4. Evidence Appraisal and Weighting

Evidence was appraised narratively rather than through formal risk-of-bias scoring. Greater interpretive weight was given to longitudinal human studies, pregnancy and postpartum cohort studies, studies directly measuring vaginal glycogen, pH, *Lactobacillus* dominance, or inflammatory markers, and strain-resolved genomic or functional studies of key vaginal taxa. Mechanistic animal studies and in vitro experiments were used to support pathway plausibility but were not treated as direct evidence of human clinical effect. Candidate-gene and genome-wide association studies were interpreted cautiously, especially when findings were population-specific, observational, or not replicated across cohorts.

Particular attention was given to the distinction between established findings and inferential extensions. For example, the relationship among estrogenic state, epithelial glycogen, *Lactobacillus* dominance, and low vaginal pH was treated as a relatively well-supported biological pathway. In contrast, direct links between specific human host genotypes and VMB stability were treated as emerging and hypothesis-generating. To distinguish clinical evidence from inference, candidate features were categorized as supported by direct human vaginal evidence, limited human associative evidence, or speculative evidence inferred from animal, in vitro, or non-vaginal studies. This distinction informed the proposed conceptual hierarchy.

### 2.5. Synthesis Approach

The synthesis proceeded in four stages. First, evidence was organized around endocrine regulation of the vaginal mucosa, with emphasis on pregnancy, postpartum transition, lactation, menopause, and other hypoestrogenic states. Second, studies of glycogen metabolism, bacterial carbohydrate utilization, *Lactobacillus*-associated lactic acid production, and vaginal pH regulation were integrated to define the estrogen–glycogen–*Lactobacillus* axis as a central substrate-control pathway. Third, microbial genomic evidence was reviewed to identify strain- and species-level functional traits that may support stability or dysbiosis, including glycogen degradation, lactate production, mucin degradation, biofilm persistence, and inflammatory activation. Fourth, evidence concerning candidate host genetic, epithelial, and immune pathways was integrated with endocrine and microbial evidence to develop a systems-level conceptual hierarchy of vaginal ecological resilience.

This staged synthesis supported the development of a proposed conceptual multi-omic hierarchy. The hierarchy organizes endocrine state, candidate host modifiers, microbial functional capacity, immune tone, and reproductive phase as interacting domains that may influence VMB resilience. It was not derived from original patient data, statistically validated, or intended to produce a probability score. It is presented as a hypothesis-generating framework for future longitudinal testing.

### 2.6. Limitations of the Review Method

As a narrative review, this article does not claim exhaustive retrieval of all available studies and does not provide pooled effect estimates. The synthesis is interpretive and mechanism-oriented, which is appropriate for theory development but introduces the possibility of selection bias. In addition, several components of the proposed conceptual hierarchy depend on indirect evidence, including extrapolation from animal models, non-vaginal tissue studies, in vitro microbial experiments, and broader systems-biology frameworks. Therefore, the hierarchy should be interpreted as hypothesis-generating and requires longitudinal, multi-omic validation before any quantitative or clinical application.

## 3. Endocrine Regulation and Candidate Genetic Modifiers of the Reproductive Tract

The vaginal mucosa is a hormonally responsive epithelial barrier whose structure, mucus, glycogen, and immune environment shape vaginal microbial balance [1]. Host epithelial, metabolic, and immune signaling pathways translate endocrine signals into local programs that regulate epithelial integrity, glycogen availability, pH, and microbiota composition [1].

Preclinical mouse evidence indicates that epithelial ESR1 links estradiol signaling to vaginal maturation, glycogen accumulation, luminal pH, and microbial composition [16]. In humans, high-estrogen states such as pregnancy are associated with increased epithelial glycogen, lower vaginal pH, and Lactobacillus-dominant communities [1,17]. These findings support a conceptual model in which estrogen-responsive epithelial pathways influence metabolism, barrier function, mucosal immunity, and microbial ecology [1,16,17]. At a systems level, hormones can be viewed as physiological inputs whose effects are mediated through receptor signaling and downstream metabolic and immune pathways. Accordingly, postpartum estrogen withdrawal, menopausal decline, and iatrogenic suppression may alter epithelial function and vaginal ecological stability; whether common host genetic variants materially modify these responses remains unresolved.

### 3.1. Estrogen Receptor Signaling as a Regulatory Module in the Lower Reproductive Tract

In the lower reproductive tract, ESR1 and ESR2 form a distributed epithelial, stromal, and immune signaling axis linking estradiol to epithelial differentiation, barrier integrity, mucus and glycogen production, and mucosal immune tone [1,18]. Associations of ESR1 XbaI and ESR2 2681–4A>G polymorphisms with hypospadias risk have been reported [19], but these findings concern a different tissue and phenotype and do not establish genotype-dependent effects on vaginal mucosal or microbiome responses. Experimental studies of cervicovaginal epithelium indicate that estradiol–ESR1 signaling remodels surface architecture and CD44/CD47 expression, with implications for neutrophil trafficking and luminal antimicrobial defense [18,20].

Again, in mice, epithelial ESR1 loss impairs vaginal epithelial maturation, reduces glycogen, raises pH, and shifts microbiota toward higher-pH communities depleted of Lactobacillus, positioning ESR1 as a regulator of glycogen-dependent acidification and microbial stability [16]. These data help support a role for epithelial ESR1 in coordinating barrier structure, neutrophil access, and metabolic conditions in mice; the effects of common human ESR1/ESR2 polymorphisms on vaginal hormone responsiveness remain unknown.

### 3.2. Glycogen Metabolism and Systemic Substrate Control

Estrogen-dependent vaginal epithelial maturation increases glycogen accumulation, which is mobilized into carbohydrates that fuel lactic-acid-producing lactobacilli [12,21,22]. Glycogen turnover links endocrine state to microbial energy supply. Estradiol sustains a glycogen-rich epithelium, and exfoliated glycogen is cleaved by host α-amylase into carbohydrates that support lactobacilli, reinforcing low pH and community dominance [21,22]. Empiric data from human studies support the idea that endocrine inputs and glycogen availability jointly constrain this substrate pool. For example, longitudinal sampling showed that cell-free glycogen varied across women and over time and was strongly inversely associated with vaginal pH, consistent with glycogen supporting more acidogenic communities [23]. In postmenopausal women, low-dose estradiol increased vaginal maturation, lowered pH, and shifted communities toward *Lactobacillus* dominance [24]. These findings support the estrogen–glycogen–*Lactobacillus* axis as a substrate-control system in which estradiol influences glycogen availability and the microbial energy landscape. Although studies in other tissues identify genetic regulation of glycogen synthesis and glucose transport [13,14,25], direct evidence that these variants influence vaginal substrate handling, dysbiosis risk, or the stability of Lactobacillus-dominant states is lacking.

### 3.3. Candidate Genetic Modifiers of the Estrogen–Mucosa Network

Beyond local metabolism, CYP19A1 and SHBG variants have been associated with circulating estradiol, estradiol–testosterone balance, SHBG levels, and estradiol–SHBG ratios in postmenopausal women [10]. These associations demonstrate effects on systemic hormone phenotypes but do not establish effects on vaginal mucosal function or microbiome stability. In the proposed conceptual hierarchy, hormonal state is therefore treated as an established physiological input, whereas host variation in CYP19A1, SHBG, ESR1/ESR2, and related loci is considered a candidate modifier rather than a validated determinant of vaginal ecology [10,15,19]. Direct longitudinal human studies are needed to determine whether these variants measurably alter vaginal ecological responses to hormonal change.

### 3.4. Temporal Dynamics: Pregnancy, Postpartum, and Lactation

Across the reproductive life course, endocrine inputs move through distinct temporal regimes with characteristic mucosal consequences. During pregnancy, placental estrogens rise to sustained high levels and are consistently associated with thickened vaginal epithelium, low pH, and communities dominated by *Lactobacillus* with low alpha diversity [4,17]. These *Lactobacillus*-rich, low-entropy states are thought to be supported by increased epithelial glycogen and its downstream carbon pools, although glycogen has been inferred more often than directly quantified in pregnancy [4,12].

The early postpartum estrogen drop is an ecological perturbation. That is because within six weeks, many women shift from pregnancy-like, *Lactobacillus*-dominant profiles to more diverse, BV-associated anaerobic communities [4]. This transition is consistent with reduced estrogen-driven epithelial maturation and glycogen support, which may increase susceptibility to reproductive-tract dysbiosis and inflammation [17]. Across reproductive-age and pregnancy cohorts, high-diversity, Lactobacillus-depleted communities have been associated with genital inflammation, HIV acquisition, and preterm birth [3,7,18]. These observations support viewing postpartum estrogen withdrawal as a perturbation associated with transition from a Lactobacillus-dominant state toward more diverse and potentially inflammatory communities, although direct inflammatory profiling during normal puerperium remains limited.

Animal models and human observational studies support estrogen-dependent regulation of epithelial substrates and vaginal pH across these phases. In mice, epithelial ESR1 deletion lowers vaginal glycogen, raises pH, and shifts the microbiota from Lactobacillus-dominant toward mixed communities, identifying ESR1 as a regulator of glycogen availability and community structure [16]. In women, higher genital glycogen is strongly inversely associated with vaginal pH and aligns with *Lactobacillus* dominance, though short-term sampling has not shown a simple linear relationship with serum estradiol [23]. In that sense, estradiol should be treated as an upstream contextual influence rather than a direct linear proxy for local glycogen availability or lactate production. Importantly, no studies directly link human ESR1, ESR2, or glycogen-metabolism variants to pregnancy or postpartum microbiome stability; their role in shaping setpoints and recovery trajectories remains mechanistically plausible but unproven.

Lactation creates a quasi-steady hypoestrogenic state. Prolactin suppresses ovarian function, and breastfeeding has been linked to elevated vaginal pH and low maturation index in the puerperium [26]. Also, local hyaluronic acid has been shown to improve pH and epithelial maturation, suggesting reduced estrogenic input constrains epithelial turnover and likely substrate supply, though glycogen and microbiota were not measured [26]. Pregnancy, early postpartum, and lactation therefore represent distinct endocrine regimes, comprising a high plateau, sharp decline, and sustained low plateau, respectively, that are associated with different epithelial, pH, immune, and microbial configurations [4,18]. This temporal structure provides a basis for testing whether candidate host variation in hormone signaling or substrate handling contributes to individual resilience or vulnerability to dysbiosis.

## 4. Glycogen–Estrogen Axis: A Molecular Interface

The glycogen–estrogen axis provides a molecular interface linking systemic hormonal signals to local ecological conditions in the VMB. High-estrogen states promote stratified squamous epithelial maturation and are associated with higher glycogen, lower vaginal pH, and *Lactobacillus*-dominant communities [12]. As superficial epithelial cells exfoliate or lyse, intracellular glycogen is released into the vaginal lumen, where it becomes available to host α-amylase. Ex vivo data show genital α-amylase converts glycogen into oligosaccharides metabolizable by vaginal lactobacilli, linking estrogen-supported glycogen stores to lactic acid production, low pH, and *Lactobacillus*-rich communities [12,22].

Quantitative analyses of genital secretions reinforce this substrate-mediated linkage. In undiluted vaginal fluid, cell-free glycogen can reach percent-level concentrations and varies widely between women [23]. Higher glycogen is strongly inversely associated with vaginal pH [23] and is more common in *Lactobacillus*-dominant communities than in diverse, anaerobe-rich states [12,27]. Glycogen and amylase-derived products therefore provide a biochemical bridge between endocrine states that shapes the energy landscape available to vaginal microbes. However, vaginal glycogen availability is not explained by circulating hormone levels alone [23]. Variants in CYP19A1 and COMT have been associated with circulating estrogen and estrogen-metabolite profiles in postmenopausal women [15], and GYS1 encodes a glycogen-synthesis enzyme regulated by phosphorylation and glucose-6-phosphate activation [13,14]. Admittedly, these findings derive primarily from systemic or non-vaginal contexts and do not establish effects on vaginal epithelial glycogen or microbiome stability. The glycogen–estrogen axis is therefore treated as a supported physiological pathway, whereas host genetic variation in hormone metabolism and glycogen handling is considered a candidate modifier requiring direct human vaginal validation.

### 4.1. From Epithelial Glycogen to Bacterial Substrate: Enzymology In Situ

Vaginal epithelial glycogen must be converted into smaller transportable sugars before resident bacteria can metabolize it. Human α-amylase in cervicovaginal secretions hydrolyzes epithelial glycogen into maltose, maltotriose, and related malto-oligosaccharides [22]. In vitro and ex vivo assays show that many genital *Lactobacillus* isolates rely on α-amylase-processed glycogen products, linking host glycogen metabolism to the nutritional ecology of vaginal lactobacilli [22]. This process converts estrogen-supported epithelial glycogen into fermentable substrates that sustain lactic acid production and a low-pH, *Lactobacillus*-dominant niche.

Vaginal microbes also contribute glycogen-degrading enzymes: PulA-like pullulanases have been identified in *Gardnerella vaginalis*, *L. crispatus*, *Lactobacillus iners*, *Mobiluncus mulieris*, and *Prevotella bivia* [28,29]. PulA enzymes cleave glycogen and related α-glucans into maltodextrins, supporting glycogen growth of amylase-deficient *L. crispatus* on glycogen and creating cross-fed substrates within the community. PulA homologues differ in substrate preference and pH range, with several remaining active at the acidic pH typical of *Lactobacillus*-dominated vaginal environments (~3.5–5.5). Multi-omics data show these genes are present and expressed across community state types, indicating glycogen breakdown persists despite shifts in community composition [28]. Host α-amylase and bacterial enzymes form a distributed glycogen-processing network that sustains fermentable oligosaccharides and grounds the glycogen–estrogen axis in active luminal metabolism [1].

### 4.2. Microbial Genetic Programs for Glycogen Catabolism and Uptake

Within *Lactobacillus*-dominant communities, *L. crispatus* appears suited to use host-derived glycogen. Earlier work suggested vaginal lactobacilli rely mainly on host α-amylase for fermentable glycogen products [22,27], but direct evidence shows most clinical *L. crispatus* isolates can grow on extracellular glycogen alone [29]. Comparative genomics of 33 vaginal *L. crispatus* isolates identified a conserved gene for a putative cell-surface GH13 type I pullulanase, with domains consistent with extracellular glycogen processing [29]. Strains with N-terminal pullulanase deletions showed reduced or absent glycogen growth, whereas intact N-termini supported more efficient glycogen use [29]. Strain-level variation in this glycogen-degrading enzyme may shape *L. crispatus* glycogen use and in vivo dominance through PulA-like protein integrity and surface display.

Other vaginal taxa also encode glycogen-degrading capacity. *Gardnerella* spp. secrete extracellular α-amylase and amylopullulanase that cleave glycogen into malto-oligosaccharides, reinforcing glycogen breakdown as a community process [30]. *Gardnerella* GH13 enzymes produce oligosaccharides similar to host pancreatic α-amylase, suggesting host and bacterial enzymes converge on a shared diffusible substrate pool [30]. Genomic surveys show that several vaginal species, including *L. crispatus*, *L. iners*, and *L. gasseri*, encode amylases, pullulanases, glycosidases, and sugar transporters needed to process and metabolize glycogen-derived sugars [29,30]. And host α-amylase [22] and microbial genetic programs form a modular pathway from epithelial glycogen to intracellular carbon flux, with strain- and species-specific enzyme repertoires shaping participation in glycogen catabolism.

### 4.3. Candidate Host Genetic Modifiers of Substrate Supply

Empirical work shows that luminal glycogen varies widely among women and is more closely associated with vaginal ecology than circulating estradiol. In undiluted genital fluid, cell-free glycogen ranges from 0.1 to 32 μg/μL, is strongly inversely associated with vaginal pH, and shows no association with serum estradiol [23]. In a longitudinal cohort of Black adolescent women, glycogen was higher in *Lactobacillus*-dominant communities, unrelated to estradiol or stress, and associated with lower pH and Nugent scores and greater abundance of *L. crispatus* and *L. jensenii* [27]. These findings indicate that vaginal glycogen was more closely associated with community composition than serum estradiol in the studied cohorts, while leaving the sources of interindividual variation unresolved. These studies suggest that endocrine state supports epithelial glycogen production, whereas local host and microbial processes influence the glycogen available to the microbiota. Direct human evidence linking host genotypes to vaginal glycogen levels or microbiome states is lacking; findings from other tissues therefore provide hypotheses rather than validated vaginal mechanisms.

GYS1 encodes glycogen synthase, a central enzyme in skeletal muscle glycogen synthesis; an intronic XbaI polymorphism has been linked to reduced enzyme activity, metabolic disease traits, type 2 diabetes, and increased cardiovascular mortality in men [13]. Structural and biochemical studies show that GYS1 activity is regulated by phosphorylation and glucose-6-phosphate activation, indicating that modest regulatory changes can meaningfully alter glycogen flux [14], but these studies do not address vaginal epithelial glycogen. Similarly, SLC2A1-encoded GLUT1 is broadly expressed and mediates basal glucose uptake across many cell types. Mutations and regulatory variants in SLC2A1 alter GLUT1 expression or function and are linked to impaired glucose availability and diabetic microvascular complications [25]. These genes therefore identify candidate pathways for investigation, but current evidence does not establish that their variants alter vaginal epithelial substrate availability.

By extension, GYS1, SLC2A1, and glycogen-degradation pathways are candidate targets for future investigation of vaginal substrate supply. No human study has shown that variants in these genes preserve vaginal glycogen during hormonal decline, alter vaginal pH, or affect anaerobe expansion. Their inclusion in the conceptual hierarchy should therefore be understood as hypothesis-generating and separate from the direct evidence that vaginal glycogen is associated with low pH and Lactobacillus dominance [23,27].

### 4.4. Lactic Acid, pH Regulation, and Community Stability

Once glycogen-derived maltodextrins enter *Lactobacillus* metabolism, lactic acid becomes a major ecological effector. In health-associated communities, lactobacilli produce most vaginal lactic acid, generating both L- and D-lactate, while epithelial cells contribute only a minor fraction of L-lactate [12]. At physiological concentrations of approximately 100 mM, undissociated lactic acid contributes to a cervicovaginal pH below 4 and has demonstrated antimicrobial activity in experimental studies against BV-associated and opportunistic organisms, including *G. vaginalis*, *Trichomonas* vaginalis, *Neisseria* gonorrhoeae, *Chlamydia* trachomatis, HSV, and HIV, while acidic *Lactobacillus*-dominant states are also associated with reduced HPV persistence [12,18,31,32]. Protective effects vary by lactate isomer production: *L. crispatus*, *L. gasseri*, and *L. jensenii* produce both L- and D-lactic acid, whereas *L. iners* produces only L-lactic acid and lacks D-lactate and hydrogen peroxide production [12]. D-lactic acid appears especially protective: levels are highest in *L. crispatus*-dominant communities, lowest in *L. iners*-, *Gardnerella*-, or streptococci-dominant states, and associated with lower pH and fewer dysbiosis markers [12]. These functional distinctions parallel the ecological behavior of different community state types. *Lactobacillus*-dominant communities, especially *L. crispatus*, are linked to lower genital inflammation, reduced HIV acquisition and preterm-birth risk, and greater stability, whereas *L. iners*- or anaerobe-dominant states are more variable and BV-prone [4,12,18]. Within the glycogen–estrogen framework, L- and D-lactic acid production by *L. crispatus* and related species supports a stable low-pH, antimicrobial state, whereas *L. iners*-dominant communities, lacking D-lactate, appear to occupy less stable ecological states that are more prone to higher pH and anaerobe expansion.

### 4.5. Endocrine Phase Windows and Glycogen–Microbiome Dynamics

Across reproductive phases, shifts in the sex-steroid milieu reshape conditions that support or erode *Lactobacillus*-dominant communities. During pregnancy, high placental estrogens, especially estradiol and estriol, are associated with increased *Lactobacillus* abundance, lower vaginal pH, and reduced microbial diversity [4,5]. After delivery, estrogens fall sharply, and many women shift within weeks to months from pregnancy-associated *Lactobacillus* dominance to more diverse, BV-associated anaerobic communities enriched in *G. vaginalis* and *P. bivia* [4,5]. This postpartum window is also associated with higher vaginal pH and greater susceptibility to genital tract dysbiosis [5,6]. In breastfeeding women, lactation sustains hypoestrogenism through prolactin-mediated ovarian suppression and is associated with elevated vaginal pH and a low maturation index in early puerperium [26].

Although direct vaginal glycogen data across these phases remain limited, the estradiol–glycogen–*Lactobacillus* framework supports viewing pregnancy, postpartum, and lactation as distinct substrate contexts. High-estrogen pregnancy is consistent with a thick, glycogen-rich epithelium, whereas postpartum estrogen withdrawal and lactational hypoestrogenism may limit glycogen renewal and reduce carbon supply for lactobacilli. This interpretation aligns with data showing that glycogen, not estradiol alone, is the more proximate correlate of low pH and *Lactobacillus* dominance, with higher glycogen linked to lower pH, lower Nugent scores, and enrichment of *L. crispatus* and *L. jensenii* [23,27]. Interindividual differences in local substrate production and consumption may influence ecological responses to endocrine change. However, the contribution of specific host genetic variants is unknown, and no ESR1 or glycogen-pathway genotype has been linked directly to pregnancy or postpartum microbiome trajectories. Table 1 therefore distinguishes directly observed vaginal mechanisms from candidate host pathways extrapolated from other tissues.

## 5. Microbial Genetics in Hormone-Structured Niches

The VMB exists within a hormonal landscape that shifts across menstruation, pregnancy, postpartum, and menopause. Estrogen and progesterone fluctuations reshape epithelial thickness, glycogen availability, mucus, and pH, altering the physical and metabolic niches available to vaginal microbes [1,12,21]. Longitudinal pregnancy and postpartum studies illustrate this pattern. High pregnancy estrogen levels are associated with low-diversity, *Lactobacillus*-dominant communities, whereas postpartum estrogen decline is followed by greater diversity and enrichment of anaerobes such as *Gardnerella* and *Prevotella* [4,5]. Across the life course, reproductive phase and gonadal-hormone status are associated with shifts in community state types and alpha diversity, positioning hormone context as an important environmental filter on the vaginal ecosystem [4,21].

Within these hormone-shaped niches, microbial genomes influence which taxa dominate, persist, or decline. Comparative genomics of vaginal *L. crispatus* isolates has identified conserved glycogen-degradation and cell-surface glycosylation genes, including a surface-anchored pullulanase whose N-terminal variants reduce glycogen-supported growth [29]. Vaginal bacteria, including *L. crispatus* and *Gardnerella* spp., encode glycogen-active enzymes that convert epithelial glycogen into shared maltodextrin pools, supporting strain growth and community cross-feeding [22,34,35]. These substrate-use programs interact with hormone-driven glycogen and pH shifts, helping explain why *L. crispatus* communities are often more stable and protective than *L. iners*-dominant or anaerobe-rich states [12,18,29]. Thus, endocrine signals define the niche, while microbial genes for substrate use, stress tolerance, and host interaction help determine which lineages persist over time.

### 5.1. Hormone–Microbe Signaling and Transcriptional Responses

Cervicovaginal bacteria encounter steroid hormones mainly through host-mediated environmental changes rather than direct hormone sensing. High- and low-estrogen states reshape epithelial structure, glycogen availability, mucus, and pH, contributing to community state type shifts across puberty, menstruation, pregnancy, and menopause [6]. Meta-analytic ordination shows Tanner II, menstruation, early pregnancy, BV-positive states, and postmenopause clustering with higher diversity and anaerobe enrichment, while mid–late puberty, reproductive-age *Lactobacillus* dominance, and late pregnancy cluster with low-diversity, *Lactobacillus*-rich CSTs [6,18]. This evidence supports viewing gonadal hormone fluctuation as a moving environmental filter that may indirectly favor specific taxa and strain-level programs.

Within BV-associated communities, *Gardnerella* often acts as a central ecological and biofilm-forming organizer. Comparative genomics showed that *G. vaginalis* carries virulence and adaptation genes, including mucin-degrading glycosidases, biofilm-associated glycosyltransferases, toxin–antitoxin systems, and resistance loci that support dense epithelial biofilms [41]. In vivo, *Gardnerella*-dominant biofilms can persist after oral metronidazole, with fluorescence in situ hybridization showing structured *G. vaginalis* and *Atopobium vaginae* biofilms re-forming within weeks despite clinical cure [42]. This suggests that antibiotics may shift symptoms and planktonic flora while leaving a biofilm reservoir adapted to the vaginal niche. Polymicrobial biofilm models further show that BV-associated taxa can tune virulence together: *A. vaginae* and *P. bivia* incorporate into pre-formed *G. vaginalis* biofilms and upregulate biofilm-maintenance and virulence-associated genes [43]. These interbacterial interactions, combined with hormone-shaped community shifts, suggest that vaginal niche condition may favor BV-associated taxa and transcriptional programs that promote adhesion, biofilm stability, and persistence [6,43].

Hormones also reshape the biochemical environment in which these transcriptional programs function. Across reproductive phases, estrogen and progesterone shifts alter glycogen availability, mucosal thickness, and pH. High-estrogen states favor glycogen-rich, *Lactobacillus*-dominant communities, whereas low-estrogen or hormonally perturbed states show higher diversity and anaerobe enrichment [6,18]. In undiluted genital fluid, free glycogen can reach ~3% *w*/*v* and is strongly inversely associated with vaginal pH; multivariable models identify glycogen, rather than serum estradiol alone, as a proximal substrate variable associated with pH [23]. In adolescents, glycogen was associated with *Lactobacillus*-dominant rather than low-*Lactobacillus* communities and correlated with lower pH and Nugent scores [27].

In vitro studies show that *L. crispatus* uses flexible genetic programs to match metabolism to available glycogen-derived substrates. Glycogen use depends on surface-exposed pulA: strains with intact pulA grow on glycogen and acidify the medium to low vaginal pH, whereas pulA-defective strains do not, and such variants are common in vivo [36]. Proteomic and enzymatic assays show pulA is carbon-regulated. Simple sugars suppress pulA and α-glucosidase activity, while glycogen or galactose induces a pulA-rich S-layer and strong glycogen-degrading capacity [36]. This supports a model in which lactobacilli adjust carbohydrate metabolism to substrate availability, sustaining growth and lactic acid production as glycogen supply fluctuates within hormone-conditioned niches.

### 5.2. Genomic Adaptations of Key Vaginal Species and Strains

Vaginal *L. crispatus* has a larger and more functionally diverse genome than *L. iners*, with broader carbohydrate, amino-acid, transport, and lactate-production capacity [29,44,45]. Its pangenome encodes enzymes for fermenting multiple glycogen-derived sugars and consistently carries both D- and L-lactate dehydrogenase genes, supporting its strong acid-producing phenotype and fitness in low-pH, glycogen-rich niches [29,45]. Strain-level data identify a key glycogen-use module: most vaginal *L. crispatus* strains carry a surface-associated type I pullulanase, while N-terminal deletions markedly impair growth on glycogen alone [29]. Pregnancy metagenomes further show conserved PulA across *L. crispatus* and *L. iners* CSTs, but a cell-surface glycan cluster containing MucBP domains, S-layer proteins, and glycosyltransferases is enriched in *L. crispatus* and largely absent from *L. iners* and *G. vaginalis* [45]. Several host-interface genes show positive selection, and this glycan cluster is associated with lower alpha diversity, suggesting that cell-surface glycosylation may support host interaction, persistence, and low-diversity community structure [29,45].

In contrast, *L. iners* has a streamlined ~1.3 Mb genome, retaining core metabolism and transport systems but lacking many carbohydrate-use, amino-acid biosynthesis, and stress-response pathways found in *L. crispatus* [40,44,46]. *L. iners* produces only L-lactate and lacks the D-lactate dehydrogenase and pyruvate oxidase found in *L. crispatus*, consistent with a narrower acidification and antimicrobial profile [40,44]. Instead, it carries niche-adaptation traits, including iron–sulfur genes, alternative sigma factors, transport and restriction–modification systems, and inerolysin [40,46,47,48]. Inerolysin is a pore-forming toxin produced by clinical isolates and active at vaginally relevant pH levels (4.5–6.0) [48]. In BV-like communities, *L. iners* upregulates inerolysin, glycogen/mucin uptake, and stress-defense genes, while expressing a more commensal metabolic program in *Lactobacillus*-dominant states, supporting its role as a context-dependent facultative commensal [40]. Species-level contrasts extend to BV-associated anaerobes: *G. vaginalis* has a highly diverse pangenome with horizontally acquired pathogenicity and resistance islands encoding mucin-degrading enzymes, vaginolysin, and stress/resistance determinants [41,47,49]. *Gardnerella* sialidases NanH2 and NanH3 remove sialic acids from mucins, secretory IgA, and group B *Streptococcus* capsules, and their presence tracks strongly with BV-associated, *Lactobacillus*-depleted communities [30]. This evidence places *L. crispatus*, *L. iners*, and BV-associated anaerobes along a functional genomic continuum—metabolically versatile acidifying commensals, streamlined stress-adapted lactobacilli, and genetically plastic pathobionts differentially suited to vaginal niche conditions.

### 5.3. Estrogen-Linked Resource Regimes and Microbial Selection

Across reproductive phases, estrogen-associated shifts in epithelial substrates and pH create resource regimes that favor different microbial strategies. During pregnancy, high placental estrogens align with low-diversity, *Lactobacillus*-dominant communities and reduced BV-associated anaerobes, consistent with a glycogen-supported, low-pH regime, although substrate availability is often inferred rather than directly measured in pregnancy cohorts [4,5,12]. This interpretation is supported by studies in nonpregnant women showing that cell-free glycogen is inversely associated with vaginal pH and positively associated with *Lactobacillus*-dominant communities, whereas serum estradiol is not a significant predictor [23,27]. Within this high-substrate niche, *L. crispatus* appears glycogen-adapted, encoding broad carbohydrate metabolism, lactate production, and a surface pullulanase that supports glycogen growth and acidification when intact [29,36]. After birth, estrogen falls sharply, and many women shift within six weeks from pregnancy-like *Lactobacillus* dominance to more diverse communities enriched in *Gardnerella*, *Prevotella*, and other anaerobes [4]. Given the glycogen–pH relationship, this postpartum transition suggests a higher-pH, less glycogen-supported regime in which *Lactobacillus* control may weaken and anaerobic generalists become more competitive. Under these permissive conditions, *G. vaginalis* and *Prevotella* spp., including *P. bivia* and *P. timonensis*, may expand using traits related to mucin and glycan degradation, amino-acid catabolism, ammonia production, biofilm-associated virulence factors, and strain-dependent antibiotic resistance [28,41].

Experimental evidence shows this balance is sensitive to small physicochemical shifts. Biogenic amines such as cadaverine, putrescine, and tyramine are elevated in CST IV and Nugent 7–10 communities and increase the likelihood of transition from *Lactobacillus*-dominant states to BV-like states over time [50]. At physiologic levels, these amines slow *Lactobacillus* growth, extend lag phases, and reduce D- and L-lactate production, weakening acidification, while spermine has more variable effects [50]. Viewed alongside glycogen–pH relationships [23,27] and pregnancy–postpartum community shifts [4], these findings are consistent with a model in which endocrine-associated substrate and pH gradients favor *L. crispatus* under glycogen-rich, low-pH conditions and *Gardnerella*/*Prevotella*-rich assemblages as acidification weakens.

### 5.4. Evolutionary and Ecological Implications of Hormone-Conditioned Niches

Recurrent endocrine cycling may create repeated ecological filters for the VMB. Across puberty, menstruation, pregnancy, postpartum, and menopause, estrogen and progesterone shifts remodel epithelial thickness, glycogen, mucus, and pH, contributing to transitions between low-diversity *Lactobacillus*-dominant states and diverse anaerobe-rich communities [21]. Over time, these repeated changes may favor microbial lineages with metabolic and regulatory plasticity across both glycogen-rich and substrate-limited conditions. Comparative genomics supports standing, phase-relevant diversity within key commensals: vaginal *L. crispatus* has a sizable pangenome with strain-level variation in surface and metabolic genes tied to the glycogen–estrogen axis [29,44,45]. Most isolates carry a surface pullulanase, but N-terminal deletions impair growth on extracellular glycogen [29]. Dysbiosis-associated strains also show altered glycosyltransferase and cell-surface glycan genes, while metagenomes identify positively selected mucin-binding and S-layer host-interface genes [29,45]. These findings are consistent with ecological filtering at the strain level, not only at the species level, according to metabolic and surface traits suited to changing hormonal and substrate conditions.

This plasticity matters for future diagnostics and microbiome-directed therapeutics because the same named taxon can behave differently across niches. *L. iners* has a streamlined ~1.3 Mb genome, relies heavily on host nutrients, and appears in both *Lactobacillus*-dominant and BV-like states, upregulating stress, defense, and host-interaction genes, including inerolysin, under dysbiosis [40,44,46]. Similarly, *G. vaginalis* includes genomospecies with distinct sialidase, vaginolysin, and resistance profiles, and greater *Gardnerella* strain diversity is linked to symptomatic BV and short-term recurrence [49]. Through regulatory rewiring, metabolic specialization, and host-interface diversification, vaginal microbes respond to substrate and niche conditions in ways that may shape stability or dysbiosis risk. A strain-resolved, hormone-contextualized framework may help identify communities requiring closer longitudinal monitoring during pregnancy and postpartum, but predictive or therapeutic use requires validation in prospective human cohorts.

## 6. Immune Modulation and Host Candidate Defense Variants

### 6.1. Overview: The Mucosal Immune Layer of Ecological Control

The vaginal mucosa is a sentinel interface where epithelial tolerance, pattern recognition, and microbial communities must remain balanced. High-diversity, *Lactobacillus*-depleted BV communities are immunologically active, with elevated pro-inflammatory cytokines, activated antigen-presenting and CD4^+^ T cells, and enriched innate immune pathways [18]. These observations suggest that dysbiosis involves altered mucosal immune calibration rather than absence of immune activity. Biogenic amines enriched in CST IV/BV-like communities, including cadaverine, putrescine, and tyramine, are associated with transition from *Lactobacillus* dominance to BV and can slow *Lactobacillus* growth while reducing D- and L-lactate production [50]. By weakening lactic-acid pH control, these metabolites may create conditions more permissive to BV-associated anaerobes and inflammatory activation [50]. Candidate host immune variants may further influence this calibration. Polymorphisms in pattern-recognition and cytokine pathways, including toll-like receptors (TLRs) and IL-1 family genes, have been associated with BV or altered genital cytokine responses in selected cohorts [18,51,52]. A genome-wide analysis in pregnant women identified 72 host variant associations with vaginal bacterial traits, including IFIT1 rs303212, which was linked to Actinobacteria and Bifidobacteriaceae abundance and showed suggestive associations with *Lactobacillus*, *G. vaginalis*, and beta diversity [11]. These findings raise the possibility that interferon-regulated immune pathways contribute to pregnancy-associated microbial composition, although causal direction remains unresolved.

These microbial, metabolic, and genetic associations support considering mucosal immunity as an interacting layer in the vaginal ecosystem. The mucosa interprets microbial and metabolite cues through pattern-recognition and interferon-linked pathways, some of which may be influenced by host genotype [11,18,51,52]. However, current evidence is insufficient to define patient-specific immune set points or genetically determined stability thresholds. Subsequent sections therefore examine pattern-recognition receptor (PRR) polymorphisms and related host-defense variants as candidate modulators of mucosal responsiveness rather than validated predictors of VMB stability.

### 6.2. Pattern-Recognition Receptor Polymorphisms and Microbial Sensing

TLRs are reproductive-tract pattern-recognition receptors that detect microbial ligands and activate NF-κB-dependent cytokine responses [18]. In HIV-1-positive African American adolescents, TLR4 rs4986790, TLR9 rs187084, and TLR2 rs1898830 variants were associated with higher incident BV risk under Nugent, Amsel, or combined diagnostic criteria [51]. Because functional assays were not performed, these findings support association rather than mechanistic causation. Sex steroids further modulate innate sensing. Estradiol and progesterone shape reproductive-tract immune responsiveness and inflammatory tone, influencing mucosal defense and susceptibility to dysbiosis [5,18]. Whether TLR genotype and endocrine phase jointly affect vaginal immune responsiveness has not been tested directly. Thus, TLR polymorphisms should be treated as candidate modifiers of mucosal sensing rather than established determinants of BV transition. After TLR activation, cytokine and antimicrobial pathways shape response magnitude and duration: Lactobacillus-depleted BV-like communities are linked to higher genital inflammation and innate immune-pathway enrichment, whereas Lactobacillus-dominant states show lower inflammatory tone [18]. This evidence suggests that PRR variation may contribute to interindividual differences in inflammatory responses, but prospective multi-omic cohorts are needed to test whether these variants predict symptomatic BV, recurrence, or recovery.

### 6.3. Cytokine and Antimicrobial Gene Variants

Cytokine and innate immune variants also shape the vaginal inflammatory set point. The IL-1β axis is strongly linked to BV-associated dysbiosis. In pregnant women, *G. vaginalis* and anaerobic Gram-negative rods are associated with increased local IL-1β and IL-1ra [17,52]. This response may be genotype-associated, as TLR4 896G carriers showed blunted IL-1β responses despite higher *G. vaginalis* levels, suggesting host polymorphisms can alter mucosal inflammatory equilibrium [52]. These findings suggest that host polymorphisms may alter mucosal inflammatory responses, but they do not establish a generalizable genotype-to-VMB mechanism. Because IL-1β is central to BV-associated inflammation, variants that alter IL-1 pathway activity remain plausible candidate modifiers of mucosal inflammatory tone. However, whether inherited cytokine variants sustain, resolve, or destabilize dysbiosis in longitudinal human cohorts remains unproven.

Antimicrobial and innate immune pathways are also relevant to dysbiosis risk. Variants in TLR and interferon-related genes have been associated in select cohorts with vaginal bacterial traits, BV susceptibility, or local cytokine responses [11,51,52]. These associations suggest candidate mechanisms involving microbial recognition, cytokine responsiveness, and antimicrobial activity, but clinical effect sizes, reproducibility across ancestry groups, and interactions with endocrine phase remain incompletely defined. Immune outcomes also depend on epithelial barrier integrity. Immune signaling interacts with mucosal architecture, influencing whether microbial communities remain luminal or disrupt the epithelial boundary. Because estradiol supports epithelial cohesion while BV-associated bacteria can weaken barrier function, epithelial pathways and endocrine crosstalk are important to host–microbe boundary maintenance. This leads into the next section on epithelial barrier pathways and endocrine signaling.

### 6.4. Epithelial Barrier Genes and Hormonal Crosstalk

Epithelial integrity in the lower reproductive tract is regulated by epithelial differentiation, mucins, and sex-steroid signaling. The stratified squamous epithelium and mucins such as MUC4 and MUC5B form a primary physical and biochemical defense layer [18]. Estradiol supports this barrier by promoting epithelial proliferation, glycogen synthesis, and epithelial thickening, whereas estrogen withdrawal during postpartum and menopausal states is associated with epithelial thinning, higher pH, and reduced *Lactobacillus* stability [17,24]. Dysbiosis-associated taxa can also weaken barrier integrity. *G. vaginalis*, *A. vaginae*, and *P. bivia* produce sialidases, cytolysins, and proteases that degrade mucins, disrupt adhesion, and increase epithelial permeability [17,30,41]. Cytolysins such as vaginolysin and inerolysin can further damage epithelial membranes and weaken junctional integrity, creating a more permeable mucosa that may support persistence of anaerobe-rich communities [17].

Hormonal context modifies epithelial vulnerability. High-estrogen states are associated with epithelial maturation, mucus production, glycogen availability, and *Lactobacillus*-mediated acidification, whereas low-estrogen states may weaken these defenses and may facilitate BV-associated anaerobe colonization [17]. Barrier-related host genetic variation is a plausible candidate modifier, but direct evidence linking such variants to VMB resilience is limited. These layers operate as an integrated system, as barrier integrity, cytokine regulation, microbial sensing, and endocrine state interact with microbial metabolism and local pH. Dysbiosis may therefore reflect disrupted coordination across immunologic, epithelial, microbial, and hormonal processes rather than a single defect. This supports viewing the vaginal ecosystem as a coupled immune–endocrine–microbial interface.

### 6.5. Immune–Endocrine Contributions to Vaginal Ecological Stability

Functionally, the vaginal ecosystem can be conceptualized as a host–microbe system linking endocrine state, innate immunity, epithelial repair, and microbial metabolism. Estradiol-associated epithelial maturation, glycogen availability, acidification, and Lactobacillus dominance are generally compatible with lower-inflammatory mucosal conditions, whereas estrogen withdrawal, epithelial disruption, or anaerobe enrichment may create conditions in which inflammatory activation and dysbiosis are more likely [17,18,24]. Pattern-recognition, cytokine, antimicrobial, and epithelial-barrier pathways may influence the magnitude and resolution of mucosal responses. However, available human data are not sufficient to assign individual immune gain, damping coefficients, or genetically defined stability basins.

Accordingly, loci such as TLR2, TLR4, IL1B, DEFB1, MBL2, and IFIT1 should be considered candidate features for future longitudinal studies rather than validated parameters for clinical prediction. Future cohorts should test whether endocrine phase, vaginal glycogen and pH, microbial functional markers, and local immune mediators interact with host variants to predict recurrence, recovery, or transition [11,18,51,52]. Until such validation is available, the immunogenetic layer should be presented as a conceptual component of the proposed hierarchy, not as a quantified determinant of individual VMB resilience.

## 7. Proposed Conceptual Hierarchy of Microbiome Stability

### 7.1. Conceptual Foundation: Microbiome Stability as a Dynamic Phenotype

The VMB is better understood as a dynamic phenotype than as a fixed list of taxa. At any given time, the VMB reflects interactions among endocrine state, epithelial substrate availability, mucosal immunity, microbial functional capacity, and reproductive phase. In conceptual system terms, stability refers to the tendency of a community to remain in, or return toward, a low-diversity *Lactobacillus*-dominant configuration after perturbation. Reduced resilience refers to increased likelihood of transition toward a higher-diversity, BV-associated configuration. Here, terms such as “state transition,” “tipping point,” and “stability landscape” are used heuristically to organize biological mechanisms; they do not denote validated mathematical boundaries or patient-specific predictions.

**Figure 1:**
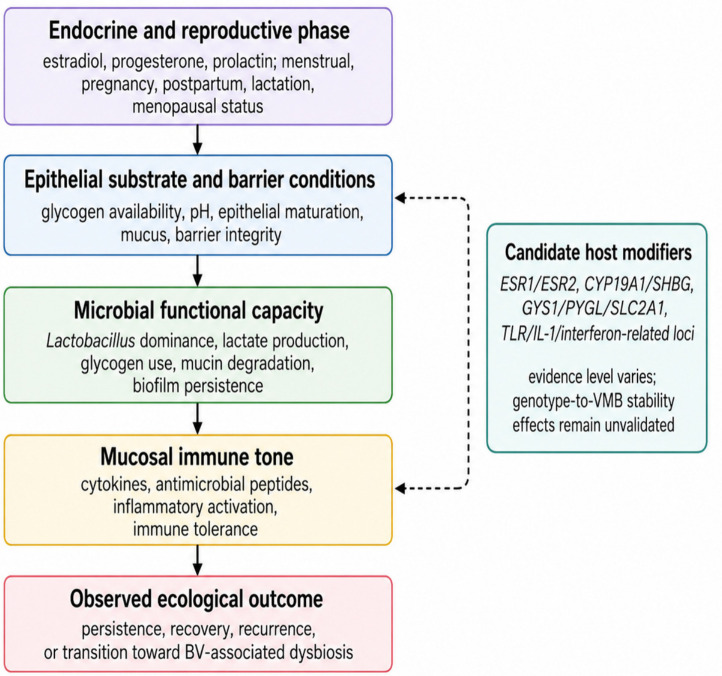
Proposed conceptual hierarchy of VMB stability. Endocrine and reproductive phase shape epithelial substrate and barrier conditions, which interact with microbial functional capacity and mucosal immune tone to influence persistence, recovery, recurrence, or transition toward dysbiosis. Candidate host modifiers are shown as hypothesis-generating features because direct human genotype-to-vaginal-microbiome stability evidence remains limited. Arrows indicate proposed biological interactions and do not represent validated coefficients, causal weights, or clinical prediction rules.

The proposed hierarchy (Figure 1) organizes candidate stability features into five interacting domains. First, endocrine context includes estradiol, progesterone, prolactin, and reproductive phase. Second, the epithelial-substrate layer includes glycogen availability, pH, epithelial maturation, and barrier integrity. Third, candidate host modifiers include hormone-signaling, glycogen-handling, glucose-transport, pattern-recognition, cytokine, and antimicrobial-defense pathways, interpreted according to their level of human vaginal evidence. Fourth, the microbial functional layer includes *Lactobacillus* dominance, strain-level carbohydrate-utilization and lactate-production modules, and BV-associated functions such as mucin degradation, sialidase activity, and biofilm persistence. Fifth, the mucosal immune layer includes cytokine tone, antimicrobial peptides, and inflammatory activation. These domains are proposed as a conceptual framework for future testing rather than as validated parameters in a predictive equation.

Future longitudinal studies may operationalize stability using empirical outcomes such as sustained low-diversity *Lactobacillus* dominance, recurrence-free intervals after treatment, recovery after postpartum transition, or persistence of low vaginal pH. However, the present review does not derive a mathematical stability function, assign feature weights, or estimate individual transition probabilities. The hierarchy is intended to identify biologically plausible feature classes that should be tested in prospective, phase-resolved human cohorts.

### 7.2. Mechanistic Domains: Substrate Availability, Lactate Production, and Ecological Stability

A low-pH vaginal environment is sustained in part by lactic acid production from lactobacilli using host- and microbe-processed glycogen-derived substrates. This lactate-supporting capacity depends on several interacting factors: endocrine support for epithelial maturation, local glycogen availability, host and bacterial glycogen-processing enzymes, abundance of lactate-producing taxa, and the presence of BV-associated organisms that consume or disrupt the low-pH niche. These relationships are biologically plausible and supported by human biochemical, microbial, and observational data, but they cannot currently be reduced to a validated human flux equation.

Therefore, lactate-supporting capacity should be treated as a mechanistic domain rather than a quantified control parameter. Serum estradiol alone is not a direct linear proxy for vaginal glycogen or lactate production, and direct evidence linking human GYS1, PYGL, or related genotypes to vaginal glycogen availability remains lacking. The conceptual hierarchy therefore separates directly measurable vaginal features, such as glycogen, pH, and Lactobacillus dominance, from candidate host genetic modifiers that require validation.

### 7.3. Multi-Omic Feature Hierarchy for Future Validation

Building on the preceding sections, the proposed framework organizes endocrine, epithelial, microbial, immune, temporal, and candidate host-modifier features that may contribute to VMB stability. Table 2 presents a candidate feature hierarchy for future empirical testing. For future model development, longitudinal cohorts would need to define stability outcomes prospectively, harmonize multi-omic measurements across sites, and test whether candidate features improve prediction beyond standard clinical and microbial variables. Until such validation is available, these feature classes should be interpreted as research priorities rather than as components of a clinical prediction tool.

Future computational models could be developed from this hierarchy only after longitudinal datasets are available with prespecified stability outcomes and harmonized measurements across endocrine, epithelial, microbial, immune, temporal, and host-modifier domains. Appropriate model development would require training and external validation cohorts, batch-effect correction, calibration assessment, uncertainty reporting, and evaluation of performance across ancestry and reproductive-phase groups. Explainability methods may be useful in future models, but no feature-attribution analysis is performed in this narrative review.

At present, the literature supports only candidate instability patterns. Examples include low vaginal glycogen or elevated pH, reduced *Lactobacillus* dominance, enrichment of BV-associated anaerobes, increased mucin-degradation or biofilm-associated functions, and elevated inflammatory cytokines. These patterns may help generate hypotheses about substrate limitation, microbial perturbation, or immune activation, but they should not be used to assign an individual patient’s dysbiosis risk to a specific hormonal, genetic, microbial, or immunologic cause without prospective validation.

From a systems-biology perspective, the proposed hierarchy provides a structured way to organize mechanisms that may influence stability. Endocrine changes alter epithelial and substrate conditions; microbial metabolism modifies pH and nutrient availability; immune signaling shapes inflammatory tone; and candidate host modifiers may affect responsiveness in ways that remain incompletely defined. These interactions justify future longitudinal and mechanistic study, but they do not yet establish a validated state-estimation variable, control parameter, Lyapunov-style metric, or clinical risk score. The revised framework therefore remains conceptual and hypothesis-generating.

## 8. Data Integration and Validation Roadmap

For future empirical testing, the proposed conceptual hierarchy would require standardized, phase-resolved collection of endocrine, epithelial, microbial, immune, temporal, and candidate host-modifier data. The workflow should begin with harmonized input acquisition across cohorts. Core measurements could include vaginal pH, vaginal glycogen or epithelial maturation indices, reproductive-phase metadata (e.g., gestational week, postpartum day, lactation status, menopausal status), targeted hormone assays where relevant (estradiol, progesterone, prolactin), local immune markers (e.g., IL-1β, β-defensin-1, MBL2), and microbial community or functional markers measured by multiplex qPCR, metagenomic sequencing, or other validated platforms. Candidate host genotyping of ESR1/ESR2, CYP19A1/SHBG, GYS1/PYGL/SLC2A1, TLR/IL-1, interferon-related, or antimicrobial-defense loci should be reserved for research cohorts and interpreted according to the evidence level assigned in Table 2.

Raw measurements should pass through a prespecified quality-control and harmonization workflow, including sample-quality assessment, extraction and assay controls, sequencing or qPCR quality thresholds, batch-effect assessment, and domain-specific normalization. This stage should prioritize cross-site reproducibility and transparent reporting of feature provenance. Because vaginal multi-omic datasets remain vulnerable to batch effects, population heterogeneity, and phase-specific sampling differences, the immediate priority is reproducible feature harmonization rather than automated computation of a probability score.

Future predictive models, if developed, should be trained only after stability outcomes are prospectively defined. Potential outcomes could include sustained low-diversity *Lactobacillus* dominance, recurrence-free intervals after BV treatment, postpartum recovery trajectories, or maintenance of low vaginal pH. Model development would require training and external validation cohorts, calibration assessment, uncertainty reporting, batch-effect correction, and evaluation of performance across reproductive phase and ancestry groups. Feature-attribution methods may be useful in future validated models, but this narrative review does not perform SHAP, integrated-gradient, or other explainability analyses and does not identify patient-specific mechanistic “fingerprints” of instability.

At the current evidence level, outputs from this framework should not be described as diagnostic dashboards, tipping-boundary estimates, clinical risk maps, or intervention thresholds. The framework should not be used to recommend patient-specific probiotics, hormonal support, antimicrobial suppression, or other preventive strategies outside prospective validation studies or established clinical indications. Its appropriate near-term role is to guide research design, feature selection, and hypothesis generation.

Validation should proceed in stages. Analytical validation should establish reproducibility of sampling, pH and glycogen measurement, qPCR or sequencing assays, genotyping, and hormone or immune measurements across laboratories. Biological validation should test whether candidate feature domains track directly measured vaginal glycogen, pH, microbial function, and local immune tone. Clinical validation should then determine whether these features predict recurrence, recovery, or transition beyond standard clinical variables and taxonomic microbiome profiles. Transportability analyses should evaluate performance across reproductive phases, ancestry groups, sites, assay platforms, and perturbation contexts.

Through this staged validation roadmap, the proposed hierarchy reframes VMB research from static taxonomic description toward integrated assessment of endocrine, epithelial, microbial, immune, temporal, and candidate host-modifier domains. However, the framework remains conceptual until longitudinal human studies demonstrate that these domains improve prediction, explanation, or management of clinically meaningful stability outcomes.

### Data Integration Architecture for Future Cohorts

Data integration should first be designed for longitudinal research cohorts rather than clinical deployment. Heterogeneous data streams can be organized into modular domains corresponding to the conceptual hierarchy. A candidate host-modifier module may include ESR1/ESR2, CYP19A1/SHBG, GYS1/PYGL/SLC2A1, TLR/IL-1, interferon-related, and antimicrobial-defense loci. These variants should not be described as static patient parameters or deterministic constraints on vaginal ecology. Instead, they should be coded as candidate features with explicit evidence levels, as summarized in Table 2.

Endocrine and epithelial-substrate modules should capture reproductive phase, hormone measurements where available, vaginal pH, glycogen availability, epithelial maturation, and barrier-related markers. The microbial module should capture community composition and selected functional markers using validated qPCR, direct-to-PCR, sequencing, or metagenomic workflows [53,54,55]. Potential microbial features include *Lactobacillus* load, *L. crispatus* versus *L. iners* dominance, *Gardnerella* diversity, BV-associated anaerobes, and functional markers such as pulA, ldhL/ldhD, nanH, and sialidase-related genes when technically validated.

An immune module may include local cytokines, antimicrobial peptides, secretory IgA, and pattern-recognition pathway markers, depending on sample type and assay feasibility. Clinical metadata, including gestational week, postpartum day, lactation status, menopausal stage, recent antibiotic exposure, sexual exposure, and hormonal therapy, should provide temporal and perturbation context. All streams should converge in a computational integration layer that produces a harmonized feature matrix for descriptive analysis, hypothesis testing, and future model development. The revised framework should not report example patient-level outputs such as “SI = 0.78” or twelve-week stability probabilities.

In aggregate, this architecture would support transparent reporting of feature domains, evidence levels, measurement uncertainty, and validation status. Rather than collapsing heterogeneous data into a single clinical score, near-term studies should determine which features are reproducible, biologically meaningful, and incrementally informative beyond standard clinical assessment and microbial taxonomy.

## 9. Research, Clinical, and Educational Implications

### 9.1. From Mechanistic Understanding to Clinical Research Translation

The convergence of endocrine physiology, microbial functional ecology, mucosal immunology, and candidate host modifiers provides a mechanistic map for studying VMB stability. Rather than revealing validated causal levers for individualized clinical care, the framework identifies biological domains that should be measured together in longitudinal cohorts. Hormone concentrations, epithelial substrate markers, microbial functional traits, immune mediators, reproductive phase, and candidate host variants should be interpreted as interacting features whose causal contributions remain to be tested. Clinically, this synthesis does not convert dysbiosis into a validated quantitative state variable. Instead, it supports a shift from taxonomic description alone toward phase-aware assessment of mechanisms associated with persistence, recurrence, recovery, or transition.

Near-term clinical relevance lies in research design and cautious interpretation rather than pre-emptive individualized intervention. Prospective studies can test whether adding vaginal pH, glycogen or epithelial maturation, endocrine phase, immune markers, strain-resolved microbial function, or candidate host modifiers improves prediction of recurrence, postpartum transition, or recovery beyond standard clinical and taxonomic variables. Host genotyping should not be used for genotype-informed counseling at present. Specific loci such as ESR1/ESR2, GYS1/PYGL/SLC2A1, TLR2, IL1B, DEFB1, MBL2, and IFIT1 should be discussed as research features requiring replication, not as validated explanations of individual stability trajectories.

In parallel, longitudinal molecular profiling may be useful in research settings for tracking microbial functional gene markers such as pulA, ldhL/ldhD, nanH, sialidase-related genes, and strain-level *Lactobacillus* or *Gardnerella* features when assays are technically validated. These markers should be interpreted alongside pH, glycogen, reproductive phase, and clinical context. At present, such integrated assessments should not be described as rendering the VMB predictable or governable. Their appropriate role is to test whether function-resolved data improve biological interpretation and future risk modeling beyond taxonomy alone.

### 9.2. Translational Research: From Taxonomy to Function

Translational research should evaluate whether endocrine, epithelial, microbial, immune, temporal, and candidate host-modifier features provide information beyond microbial taxonomy alone. The goal is not to produce a single clinical score at this stage, but to determine which measurements are reproducible, biologically meaningful, and incrementally informative for clinically relevant outcomes. At the physiological level, priority measurements include vaginal pH, glycogen availability, epithelial maturation, and barrier-related markers. These readouts provide a practical description of the local habitat in which vaginal microbes persist, recover, or transition.

A candidate host-modifier layer may include loci related to hormone signaling, substrate handling, glucose transport, pattern recognition, cytokine signaling, and antimicrobial defense. However, genotyping ESR1/ESR2, GYS1, PYGL, SLC2A1, TLR2, IL1B, DEFB1, MBL2, or IFIT1 should be limited to research contexts until replicated human studies link these variants to vaginal glycogen, pH, immune tone, microbial function, recurrence, or recovery. The microbial layer should shift emphasis from taxonomy alone to function-resolved features, including *Lactobacillus* load, *L. crispatus* versus *L. iners* dominance, *Gardnerella* diversity, BV-associated anaerobes, and functional markers such as pulA, ldhL/ldhD, nanH, and sialidase-related genes. A complementary immune layer may include local IL-1β, IL-6, TNF-α, β-defensin-1, MBL2, secretory IgA, or related markers, depending on sample type and assay feasibility.

These layers can be organized into a harmonized feature matrix for descriptive analysis, hypothesis testing, and future model development. Any future predictive model would require prespecified outcomes, training and external validation cohorts, batch-effect correction, calibration assessment, uncertainty reporting, and evaluation across reproductive phase, ancestry, and assay platform. The revised framework should not be described as producing a composite SI between 0 and 1 or a single probability of future stability.

The host–endocrine–microbial framework provides a scaffold for prioritizing validation studies across key domains of women’s health. Rather than proposing immediate diagnostic or therapeutic strategies, Table 3 identifies candidate research applications, validation endpoints, and evidence limitations that should be addressed before clinical implementation.

Educationally, the framework can help trainees and clinicians distinguish established human vaginal evidence from hypothesis-generating mechanisms. Teaching materials should emphasize that endocrine phase, vaginal pH, glycogen availability, microbial functional capacity, and mucosal immune tone are measurable domains, whereas most host-genetic features remain candidate modifiers. This distinction is essential to prevent readers from interpreting inferred mechanisms as validated clinical predictors.

## 10. Future Directions: Validation Priorities for Vaginal Microbiome Stability

### 10.1. Phase-Resolved Longitudinal Cohorts

The next step is to develop phase-resolved longitudinal cohorts that follow participants across menstrual, pregnancy, postpartum, lactation, menopausal, and treatment-related transitions. Unlike cross-sectional studies, these designs can distinguish within-person temporal change from between-person variation. Cohorts should prospectively define stability outcomes, such as sustained low-diversity *Lactobacillus* dominance, recurrence-free intervals after BV treatment, postpartum recovery trajectories, maintenance of low vaginal pH, or transition toward BV-associated dysbiosis. These outcomes would allow the proposed hierarchy to be tested empirically rather than assumed as a predictive system.

Achieving this resolution requires harmonized sampling and measurement. Cohorts should pair vaginal swabs with vaginal pH, glycogen availability or epithelial maturation indices, reproductive-phase metadata, hormone measurements where feasible, local immune mediators, and clinical perturbation variables such as antibiotics, sexual exposure, hormonal therapy, lactation, or menopausal status. Microbial profiling should include taxonomic and selected functional markers, with strain-resolved sequencing or metatranscriptomics used only when technically and analytically validated. Candidate host genotyping can be included in research protocols, but variants should be coded according to the evidence levels in Table 2.

Analyses should test whether endocrine, epithelial, microbial, immune, temporal, and candidate host-modifier features explain or predict stability outcomes beyond standard clinical variables and microbial taxonomy. Required safeguards include prespecified analytic plans, quality-control thresholds, batch-effect assessment, external validation, calibration, uncertainty reporting, missing-data handling, and evaluation across ancestry, reproductive phase, site, and assay platform.

Systems-biology and statistical tools may help characterize temporal patterns once adequate longitudinal data exist, but clinical decision-support applications would require validated dynamic models, safety evaluation, prospective clinical trials, and regulatory review.

### 10.2. Data Harmonization, Model Validation, and Intervention Readiness

Near-term computational work should focus on reproducible data integration rather than real-time clinical decision support. Future models may use conventional statistical learning or machine-learning methods to examine trajectories, but only after outcomes, feature definitions, and validation plans are prespecified. Mechanistic equations should be introduced only when the relevant parameters can be measured or estimated from human vaginal data.

Immediate standardization priorities include reproducibility of qPCR, direct-to-PCR, sequencing, and metagenomic workflows; harmonized handling of microbial functional markers such as pulA, ldhL/ldhD, nanH, and sialidase-related genes; contamination controls; platform-specific detection limits; and transparent reporting of normalization and batch-correction procedures. Molecular diagnostic and metagenomic workflows can inform assay development, but vaginal-specific analytical validation remains necessary.

Translation to intervention should be staged separately from prediction. Candidate interventions, including probiotics, antimicrobial strategies, hormonal or mucosal therapies, and future engineered microbial systems, should be evaluated in controlled studies with predefined safety, colonization, persistence, off-target, reproductive-phase, and recurrence outcomes. Engineered probiotic or controlled-release systems developed in other disease settings may be cited as technology analogs, but they do not establish feasibility for VMB control. The near-term goal is therefore to define reproducible biomarkers and validation endpoints before any clinical algorithm, closed-loop intervention, or AI-enabled decision system is proposed.

## 11. Conclusions

The narrative review reframes VMB stability as a dynamic host–endocrine–microbial phenotype rather than a static taxonomic state. The available evidence supports a central role for reproductive phase, estrogen-associated epithelial maturation, glycogen availability, vaginal pH, *Lactobacillus* dominance, microbial functional capacity, and mucosal immune tone in shaping vaginal ecological states. These interacting domains provide a biologically grounded framework for studying persistence, recovery, recurrence, and transition toward dysbiosis.

This synthesis also highlights a major evidentiary distinction. The estrogen–glycogen–*Lactobacillus* axis is supported by human physiological, biochemical, and microbiome evidence, whereas many proposed host-genetic modifiers remain inferential. Variants in estrogen signaling, glycogen metabolism, glucose transport, pattern-recognition, cytokine, and antimicrobial-defense pathways should therefore be treated as candidate features for future study, not as validated determinants of individual VMB resilience. Similarly, microbial genomic features such as glycogen-use, lactate-production, mucin-degradation, and biofilm-associated pathways provide important functional context, but their prospective predictive value requires longitudinal validation.

The proposed conceptual hierarchy is therefore best understood as a research scaffold. It can help organize future longitudinal cohorts, harmonize endocrine, epithelial, microbial, immune, temporal, and candidate host-modifier measurements, and distinguish direct human vaginal evidence from animal, in vitro, systemic, or non-vaginal inference. Before any clinical score or decision-support tool is considered, future studies must define stability outcomes prospectively, standardize multi-omic measurements, correct for batch effects, externally validate models, and evaluate transportability across reproductive phase, ancestry, site, and assay platform.

In its revised form, the framework does not claim that VMB stability can currently be computed, predicted, or clinically manipulated from multi-omic inputs. Instead, it proposes a testable hierarchy of mechanisms through which endocrine state, epithelial substrate availability, microbial function, immune tone, reproductive timing, and candidate host modifiers may interact. This restrained framing preserves the manuscript’s central contribution: moving beyond taxonomy alone while clearly identifying the validation steps required before translation to clinical prediction or intervention.

## Figures and Tables

**Table 1:** Established vaginal mechanisms and candidate host pathways in the glycogen–estrogen axis.

Level	Control Point	Core Function	Relevant Feature	Evidence-Informed Interpretation	Sources
Host endocrine signaling	ESR1, ESR2	Mediate estradiol-dependent transcription in reproductive-tract epithelial, stromal, and immune cells	Experimental ESR1 signaling in the lower reproductive tract; ESR1/ESR2 polymorphism evidence derives largely from non-vaginal phenotypes.	Candidate/indirect: receptor signaling is established, but effects of common variants on vaginal glycogen or VMB stability are unproven	[16,19,20]
Host glycogen mobilization	Glycogen-phosphorylase activity	Cleave stored glycogen into glucose-1-phosphate	Glycogen-phosphorylase activity demonstrated in cervicovaginal epithelium	May shift mucosa between glycogen-replete and glycogen-depleted states	[33]
Host glucose transport	SLC2A1/GLUT1	Regulatory variants alter GLUT1 expression or function in non-vaginal contexts	Regulatory variants alter GLUT1 expression	Speculative/inferred: a plausible substrate-supply pathway, but no vaginal genotype-to-microbiome association has been established	[25]
Host extracellular hydrolysis	Host α-amylase	Convert luminal glycogen into maltose, maltotriose, and related oligosaccharides	Vaginal α-amylase activity varies across women	Direct human vaginal biochemical evidence: generates fermentable glycogen-breakdown products that support vaginal lactobacilli growth and low-pH stability	[22,23]
Microbial glycogen degradation	pulA-type pullulanases and related glycogen-degrading enzymes	Cleave extracellular glycogen into malto-oligosaccharides	Allelic and structural variation in *L. crispatus* PulA; variable glycogen-degrading enzyme families across *Gardnerella* species	Direct isolate, genomic, and enzymatic evidence; contribution to longitudinal community stability remains to be tested	[29,34,35]
Microbial sugar transport	Trehalose-specific PTS operon	Imports trehalose and related disaccharides	Operon presence and completeness vary by strain and niche	Genomic/inferred: may enhance competitive fitness when relevant carbohydrates are available; the in vivo effect on vaginal stability remains unvalidated	[36,37]
Microbial fermentation output	ldhL, ldhD	Convert carbohydrate-derived pyruvate into L- and/or D-lactic acid	*L. crispatus*, *L. gasseri*, and *L. jensenii* produce L- and D-lactate; *L. iners* mainly produces L-lactate	Direct biochemical and human community-association evidence: supports low vaginal pH and suppression of BV-associated organisms	[12,31,32,38,39,40]

**Table 2:** Candidate multi-omic feature domains for a proposed conceptual hierarchy of vaginal microbiome stability.

Domain	Feature Classes/Examples	Functional Rationale	Level of Human Clinical Evidence	Validation Needed Before Clinical Use
Endocrine and reproductive phase	Estradiol, progesterone, prolactin; menstrual-cycle phase; gestational week; postpartum day; lactation or menopausal status; vaginal pH	Defines the hormonal and temporal context in which epithelial maturation, glycogen availability, pH, and immune tone vary	Well-established human physiologic and cohort evidence for associations with VMB states, especially pregnancy, postpartum, and hypoestrogenic states	Phase-resolved longitudinal sampling with concurrent hormone, pH, glycogen, and microbiome measures
Epithelial-substrate environment	Vaginal glycogen, α-amylase activity, epithelial maturation index, mucus/barrier markers, local pH	Provides the proximal substrate and habitat conditions that support acidification and *Lactobacillus* dominance	Direct human vaginal evidence for glycogen–pH and α-amylase–lactobacilli relationships; pregnancy/postpartum glycogen data remain limited	Direct measurement across reproductive phases and perturbations, including pregnancy, postpartum, lactation, and menopause
Candidate host modifiers	ESR1/ESR2, CYP19A1/SHBG, GYS1, PYGL, SLC2A1, TLR2, TLR4, IL1B, DEFB1, MBL2, IFIT1	Candidate pathways for hormone responsiveness, substrate handling, epithelial integrity, immune sensing, and inflammatory tone	Speculative or limited human associative evidence for VMB stability; several pathways are inferred from animal, systemic, or non-vaginal studies	Replicated longitudinal human studies linking genotype to vaginal glycogen, pH, immune tone, microbial function, recurrence, or recovery
Microbial taxonomic and functional capacity	*Lactobacillus* abundance; *L. crispatus* versus *L. iners* dominance; BV-associated anaerobes; *Gardnerella* diversity; pulA, malX, ldhL/ldhD, nanH, sialidases	Captures acidification capacity, glycogen or mucin utilization, biofilm persistence, and pathobiont expansion	Direct human community-association evidence plus strain-resolved genomic and functional evidence; prospective predictive value remains incompletely validated	Strain-resolved metagenomic, metatranscriptomic, and functional validation linked to observed transitions, recurrence, and recovery
Mucosal immune state	IL-1β, IL-6, TNF-α, β-defensin-1, MBL2, secretory IgA, PRR expression	Represents tolerance–inflammation balance, antimicrobial activity, and epithelial immune activation	Human associative evidence links BV-like states to inflammatory signatures; limited evidence for prediction of stability trajectories	Longitudinal immune profiling with simultaneous microbiome, pH, glycogen, and clinical outcome data
Temporal and perturbation context	Menstruation, pregnancy, postpartum, lactation, menopause, antibiotics, sexual exposures, hormonal therapy, probiotic or antimicrobial treatment	Provides the timing and perturbation context in which stability, transition, recurrence, or recovery is observed	Well-established for some reproductive-phase associations; perturbation-specific evidence varies by exposure and population	Dense longitudinal sampling before, during, and after defined perturbations

Note: This table distinguishes directly observed human vaginal mechanisms from candidate or inferred host pathways. The table is not a scoring instrument. Feature classes listed under “candidate host modifiers” should not be interpreted as validated clinical predictors of vaginal microbiome stability.

**Table 3:** Candidate research and validation applications of the proposed conceptual hierarchy.

Clinical or Research Context	Mechanistic Domain to Evaluate	Evidence-Appropriate Near-Term Application	Validation Endpoint	Evidence Status and Source(s)
Postpartum care	Estrogen withdrawal, epithelial maturation, glycogen availability, pH, and transition from *Lactobacillus* dominance to higher-diversity communities	Longitudinal postpartum research sampling of pH, glycogen or maturation index, microbiome composition, lactation status, and symptoms	Recovery trajectory, BV recurrence, vaginal pH, and sustained or regained *Lactobacillus* dominance	Human cohort evidence supports postpartum VMB transition; intervention guidance remains unvalidated [4,26]
Recurrent BV	Reduced *Lactobacillus* dominance, *Gardnerella* diversity, nanH/sialidase activity, biofilm persistence, and inflammatory tone	Function-resolved profiling in recurrence studies, alongside standard-of-care diagnosis and treatment	Recurrence-free interval, symptom resolution, microbial recovery, and post-treatment community stability	Human and microbial evidence supports BV-associated functional features; genotype-guided therapy remains unvalidated [8,30,42]
Fertility or IVF research contexts	Cervicovaginal inflammation, epithelial barrier markers, and dysbiosis-associated community states	Observational profiling in carefully designed reproductive cohorts; no treatment recommendation based on the present framework	Association with reproductive outcomes, inflammatory markers, and microbiome stability after adjustment for clinical covariates	Evidence is exploratory and indirect for IVF-specific decision-making [17,18]
Menopause	Hypoestrogenic epithelial thinning, elevated pH, altered epithelial maturation, and reduced *Lactobacillus* dominance	Studies evaluating established local hormonal or mucosal therapies with concurrent VMB, pH, and epithelial measurements	pH, maturation index, symptoms, and VMB composition before and after clinically indicated therapy	Human evidence supports estrogen-associated mucosal and VMB changes; microbiome-guided treatment algorithms remain unvalidated [24,56]
Pregnancy monitoring	High-diversity VMB states, preterm-birth-associated taxa, inflammation, and phase-specific microbial trajectories	Longitudinal observational monitoring of VMB composition, pH, inflammatory markers, and pregnancy outcomes	Preterm birth, ascending infection, VMB transition, and added predictive value beyond standard obstetric risk factors	Human cohort evidence links VMB diversity with adverse pregnancy outcomes; targeted preventive intervention remains unvalidated [3,7,9]
Equity and precision research	Population structure, ancestry, social determinants, reproductive phase, and candidate host variants	Transportability and calibration studies across cohorts; avoid individual SNP-based risk reports until replicated	Model calibration, subgroup performance, reproducibility, and fairness across populations	Evidence supports the need for population-aware validation; clinical genetic-risk reporting remains premature [5,11]

## Data Availability

No new data were created or analyzed in this study.
